# Prognostic value of non-insulin-based insulin resistance indices in patients with newly diagnosed coronary artery disease: a cohort study

**DOI:** 10.3389/fcvm.2026.1863508

**Published:** 2026-07-22

**Authors:** Meng Sun, Lulin Chen, Dandan Li, Xianwei Fan, Youjian Zhang, Haitao Yang

**Affiliations:** 1 Department of Cardiology, Henan Provincial Chest Hospital, Chest Hospital of Zhengzhou, Zhengzhou, China; 2 Department of Cardiology, Central China Fuwai Hospital of Zhengzhou University, Fuwai Central China Cardiovascular Hospital, Zhengzhou, China

**Keywords:** cohort study, coronary artery disease, insulin resistance, major adverse cardiovascular events, prognosis

## Abstract

**Aims:**

: Insulin resistance (IR) plays a pivotal role in the onset and progression of coronary artery disease (CAD). This study aims to assess the efficacy and clinical utility of four non-insulin-based IR indices—Triglyceride-Glucose (TyG) index, Metabolic Score for Insulin Resistance (METS-IR), Triglyceride-Glucose-Body Mass Index (TyG-BMI), and Triglyceride to High-Density Lipoprotein Cholesterol ratio (TG/HDL-C)—in predicting prognosis in patients with newly diagnosed CAD.

**Methods:**

This retrospective analysis included 890 patients with newly diagnosed CAD enrolled between January 2019 and June 2022. LASSO penalized regression was applied for covariate selection to mitigate overfitting (events-per-variable ratio ≈ 13.9), and Firth's penalized maximum likelihood estimation was performed as a sensitivity analysis. Multivariable Cox models and restricted cubic spline analyses examined associations between the four non-insulin-based IR indices and major adverse cardiovascular events (MACEs). The proportional hazards assumption was verified using Schoenfeld residual tests, and Bonferroni correction was applied for multiple comparisons (*α* = 0.0125). Receiver operating characteristic curves were plotted, and the incremental prognostic performance of each IR index for MACEs was assessed using the area under the ROC curve (AUC), C-statistics, net reclassification index (NRI), and integrated discrimination improvement (IDI). Kaplan–Meier analysis was used to evaluate the relationship between IR indices and clinical outcomes.

**Results:**

Over a median follow-up of 26.5 months, the overall incidence of MACEs was 10.9%. TyG index (HR = 1.627, 95% CI: 1.228–2.145), TyG-BMI (HR = 1.019, 95% CI: 1.014–1.023), TG/HDL-C (HR = 1.127, 95% CI: 1.055–1.195), and METS-IR (HR = 1.104, 95% CI: 1.080–1.129) were all identified as independent risk factors for MACEs. METS-IR showed the highest incremental prognostic value (C-statistic = 0.779, IDI = 0.082, NRI = 0.356, *P* < 0.001). Log-rank tests indicated significantly higher MACEs rates in patients with higher IR indices (*P* < 0.01).

**Conclusions:**

TyG index, TyG-BMI, TG/HDL-C ratio, and METS-IR are independently associated with MACEs in patients with newly diagnosed CAD, with METS-IR demonstrating the highest incremental predictive performance.

## 1 Introduction

Coronary artery disease (CAD) remains a leading cause of morbidity and mortality worldwide, contributing substantially to the global socioeconomic burden (1). In China, the incidence and mortality of CAD continue to rise due to an aging population and lifestyle shifts, particularly among middle-aged and elderly individuals, posing a major public health challenge. Reports indicate that the prevalence of CAD among individuals aged 60 and above exceeds 20%, with an increasing trend also observed in those under 40 years old (2). Collectively, these findings underscore the urgent need for improved early diagnosis and risk stratification.

Insulin resistance (IR), a hallmark of metabolic syndrome and type 2 diabetes, plays a pivotal role in the pathogenesis and progression of CAD by promoting inflammation, endothelial dysfunction, and dyslipidemia (3, 4). While the hyperinsulinemic-euglycemic clamp is the gold standard for assessing IR, its complexity and invasiveness limit routine clinical applicability (5). The Homeostatic Model Assessment (HOMA-IR) offers a simpler alternative but requires insulin measurement, restricting its feasibility in resource-limited settings (6).

These limitations have motivated the development of non-insulin-based IR indices utilizing routinely available parameters. Triglyceride-Glucose (TyG) index (7), Triglyceride-Glucose-Body Mass Index (TyG-BMI) (8), Triglyceride to High-Density Lipoprotein Cholesterol ratio (TG/HDL-C) (9), and Metabolic Score for Insulin Resistance (METS-IR) (10)—integrate key metabolic components into practical scores and have shown promising correlations with IR and cardiovascular risk (11–14). However, their prognostic value specifically in newly diagnosed CAD patients, whose metabolic state is less influenced by long-term treatments, remains underexplored. This study aims to evaluate and compare the prognostic performance of these four non-insulin-based IR indices for predicting major adverse cardiovascular events (MACEs) in patients with newly diagnosed CAD, thereby facilitating early risk stratification and personalized management.

## 2 Methods

### 2.1 Study population

**Figure 1:**
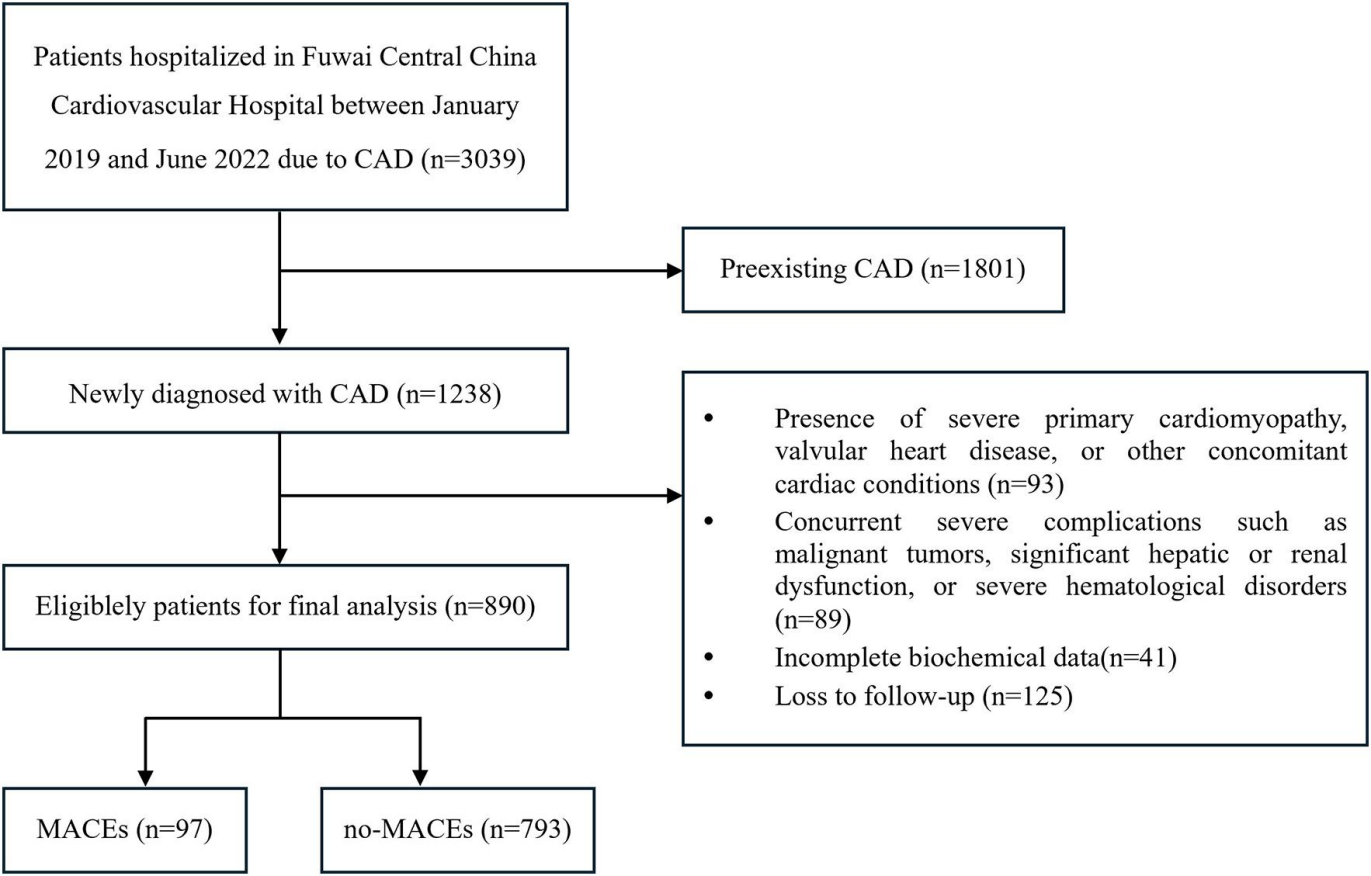
Flowchart of patient recruitment and study design.

This single-center retrospective study consecutively enrolled patients newly diagnosed with CAD at Fuwai Central China Cardiovascular Hospital between January 2019 and June 2022. CAD was defined as percutaneous coronary angiography or computed tomographic angiography examination showed that at least one coronary artery trunk or primary branch had ≥50% stenosis. The exclusion criteria were as follows: (1) presence of severe primary cardiomyopathy, valvular heart disease, or other concomitant cardiac conditions; (2) concurrent severe complications such as malignant tumors, significant hepatic or renal dysfunction, or severe hematological and endocrine disorders; (3) incomplete biochemical data or loss to follow-up. A flowchart of patient recruitment and research design is illustrated in Figure 1.

The study was approved by the Ethics Committee of Fuwai Central China Cardiovascular Hospital and conducted in accordance with the Declaration of Helsinki.

### 2.2 Data collection

All clinical data were extracted from the hospital information system. Demographic characteristics included age, sex, BMI, smoking, and drinking status. Clinical history comprised hypertension, diabetes, and prior stroke/TIA. Medication use at baseline was recorded, including antiplatelets, statins, RAS inhibitors, beta-blockers, CCBs, and antidiabetics. Of note, all enrolled patients were receiving antiplatelets therapy and statins. Laboratory parameters, measured after at least 8 h of fasting, included triglycerides (TG), total cholesterol (TC), low-density lipoprotein cholesterol (LDL-C), high-density lipoprotein cholesterol (HDL-C), fasting plasma glucose (FPG), estimated glomerular filtration rate (eGFR), serum creatinine (Cr), and serum uric acid (UA).

The four non-insulin-based IR indices were calculated as follows: (1) TyG index = Ln [TG (mg/dL) × FBG (mg/dL) ÷ 2]; (2) TyG-BMI = TyG × BMI (kg/m^2^); (3) TG/HDL-C = TG (mg/dL) ÷ HDL-C (mg/dL); (4) METS-IR = Ln [(2 × FBG (mg/dL)) + TG (mg/dL)] × BMI (kg/m^2^) ÷ Ln [HDL-C (mg/dL)]. Body mass index (BMI) was calculated as weight divided by height squared (kg/m^2^).

### 2.3 Assessment of coronary artery stenosis

Coronary stenosis severity was assessed using the Gensini score (15). The Gensini score is a widely validated and reproducible angiographic scoring system for quantifying CAD severity. Coronary vessels were divided into the left main trunk, left anterior descending artery, circumflex artery, and right coronary artery. Stenosis severity was scored as follows: <25%, 25%–49%, 50%–74%, 75%–89%, 90%–99%, and 100% stenosis were scored as 1, 2, 4, 8, 16, and 32 points, respectively. Different segments of the coronary arteries were multiplied by specific coefficients: left main trunk lesions ×5; proximal left anterior descending artery ×2.5, mid-section ×1.5, distal section ×1; first diagonal branch ×1, second diagonal branch ×0.5; proximal circumflex artery ×2.5, mid-section ×1.5, distal and obtuse marginal branches ×1, and posterior descending branch ×0.5; proximal, mid, distal segments, and posterior descending branch of the right coronary artery ×1. The total Gensini score was the sum of all segment scores.

### 2.4 Endpoints and definitions

Patients were followed via telephone or outpatient visits. The primary endpoint was major adverse cardiovascular events (MACEs), including all-cause mortality, non-fatal myocardial infarction (MI), unplanned revascularization, non-fatal stroke, hospitalization for unstable angina, heart failure, and malignant arrhythmias. Follow-up lasted from discharge until the first endpoint event or last clinical contact, with a final follow-up date of May 2024.

### 2.5 Statistical analysis

Analyses were performed using R software (version 4.3.2). Normally distributed continuous variables are expressed as mean ± standard deviation (SD), non-normal variables as median (interquartile range), and categorical variables as frequency (%). Group comparisons used one-way ANOVA, Kruskal–Wallis, Pearson's chi-square, or Fisher's exact test, as appropriate.

To mitigate overfitting given the limited number of events (*n* = 97), LASSO penalized Cox regression with 10-fold cross-validation was applied for covariate selection. Age and smoking were forced into all models based on clinical relevance. The final adjustment set comprised six covariates: age, sex, smoking, diabetes, Gensini score, and antidiabetics, yielding an events-per-variable ratio of approximately 13.9. Firth's penalized maximum likelihood estimation was performed as a sensitivity analysis to provide bias-corrected estimates. The proportional hazards assumption was assessed using Schoenfeld residual tests. Bonferroni correction was applied for multiple comparisons (*α* = 0.0125).

Variables with *P* < 0.20 in univariate analysis were included as candidates in multivariate Cox models. Multicollinearity was assessed using variance inflation factor (VIF); variables with VIF > 5 were excluded. Three Cox models were constructed: Model 1 (unadjusted), Model 2 (adjusted for age and sex), and Model 3 (additionally adjusted for smoking, diabetes, and Gensini score).

Restricted cubic splines with four knots examined non-linear relationships. AUC derived from ROC analysis assessed discriminatory ability, while C-statistic, NRI, and IDI were used to evaluate incremental prognostic performance. Optimal cut-off values were determined using the Youden index. Kaplan–Meier curves with log-rank tests compared survival between groups. *P*-value <0.05 was considered statistically significant.

## 3 Results

### 3.1 Baseline characteristics

A total of 890 patients were included, of whom 97 (10.9%) experienced MACEs during follow-up. Patients who developed MACEs had significantly higher BMI, a higher prevalence of diabetes, elevated FBG and TG levels, and lower HDL-C levels compared with those without MACEs (all *P* < 0.05). Notably, all four non-insulin-based IR indices—TyG index, TyG-BMI, TG/HDL-C ratio, and METS-IR—were significantly higher in the MACE group (all *P* ≤ 0.001), suggesting a potential role of IR in the occurrence of MACEs (Table 1).

**Table 1:** Baseline characteristics.

Variables	All (*n* = 890)	MACEs (*n* = 97)	no-MACEs (*n* = 793)	*P*-value
Age (years)	60.61 ± 10.86	62.09 ± 10.65	60.43 ± 10.88	0.150
Male	518 (58.20%)	65 (67.01%)	453 (57.12%)	0.079
BMI (kg/m^2^)	25.78 ± 3.84	28.68 ± 4.13	25.42 ± 3.65	<0.001
Smoking	205 (23.03%)	28 (28.87%)	177 (22.32%)	0.188
Drinking	331 (37.19%)	33 (34.02%)	298 (37.58%)	0.567
Hypertension	552 (62.02%)	58 (59.79%)	494 (62.30%)	0.713
Diabetes	308 (34.61%)	47 (48.45%)	261 (32.91%)	0.003
Prior stroke/TIA	102 (11.46%)	8 (8.25%)	94 (11.85%)	0.377
Gensini score	40.56 ± 20.58	46.99 ± 25.93	39.77 ± 19.71	0.009
PCI	493 (55.4%)	435 (54.9%)	58 (59.8%)	0.356
TC (mmol/L)	4.07 ± 1.15	4.00 ± 1.06	4.08 ± 1.16	0.478
TG (mmol/L)	1.59 ± 0.81	1.78 ± 0.88	1.57 ± 0.80	0.030
HDL-C (mmol/L)	1.10 ± 0.30	0.98 ± 0.26	1.12 ± 0.30	<0.001
LDL-C (mmol/L)	2.46 ± 0.91	2.34 ± 0.87	2.48 ± 0.92	0.141
FBG (mmol/L)	6.32 ± 2.64	7.20 ± 3.14	6.22 ± 2.56	0.004
eGFR (mL/min/1.73m^2^)	85.52 ± 17.54	84.31 ± 19.11	85.66 ± 17.35	0.508
Cr (µmol/L)	77.61 ± 22.18	78.96 ± 35.96	77.44 ± 19.87	0.685
UA (µmol/L)	311.18 ± 84.03	313.31 ± 78.76	310.92 ± 84.69	0.780
Medications (*n*, %)				
RAS inhibitors	249 (27.98%)	33 (34.02%)	216 (27.24%)	0.199
Beta-blockers	525 (58.99%)	63 (64.95%)	462 (58.26%)	0.248
CCBs	252 (28.31%)	30 (30.93%)	222 (27.99%)	0.878
Antidiabetics	297 (33.37%)	46 (47.42%)	251 (31.65%)	0.002
TyG index	8.78 ± 0.77	9.07 ± 0.73	8.75 ± 0.77	<0.001
TyG-BMI	226.70 ± 41.34	260.58 ± 44.97	222.56 ± 38.93	<0.001
TG/HDL-C ratio	3.61 ± 2.29	4.49 ± 2.65	3.51 ± 2.21	0.001
METS-IR	40.93 ± 7.74	48.01 ± 8.49	40.07 ± 7.19	<0.001

All patients were receiving antiplatelet agents and statins at baseline.

### 3.2 Association between non-insulin-based IR indices and MACEs

**Figure 2:**
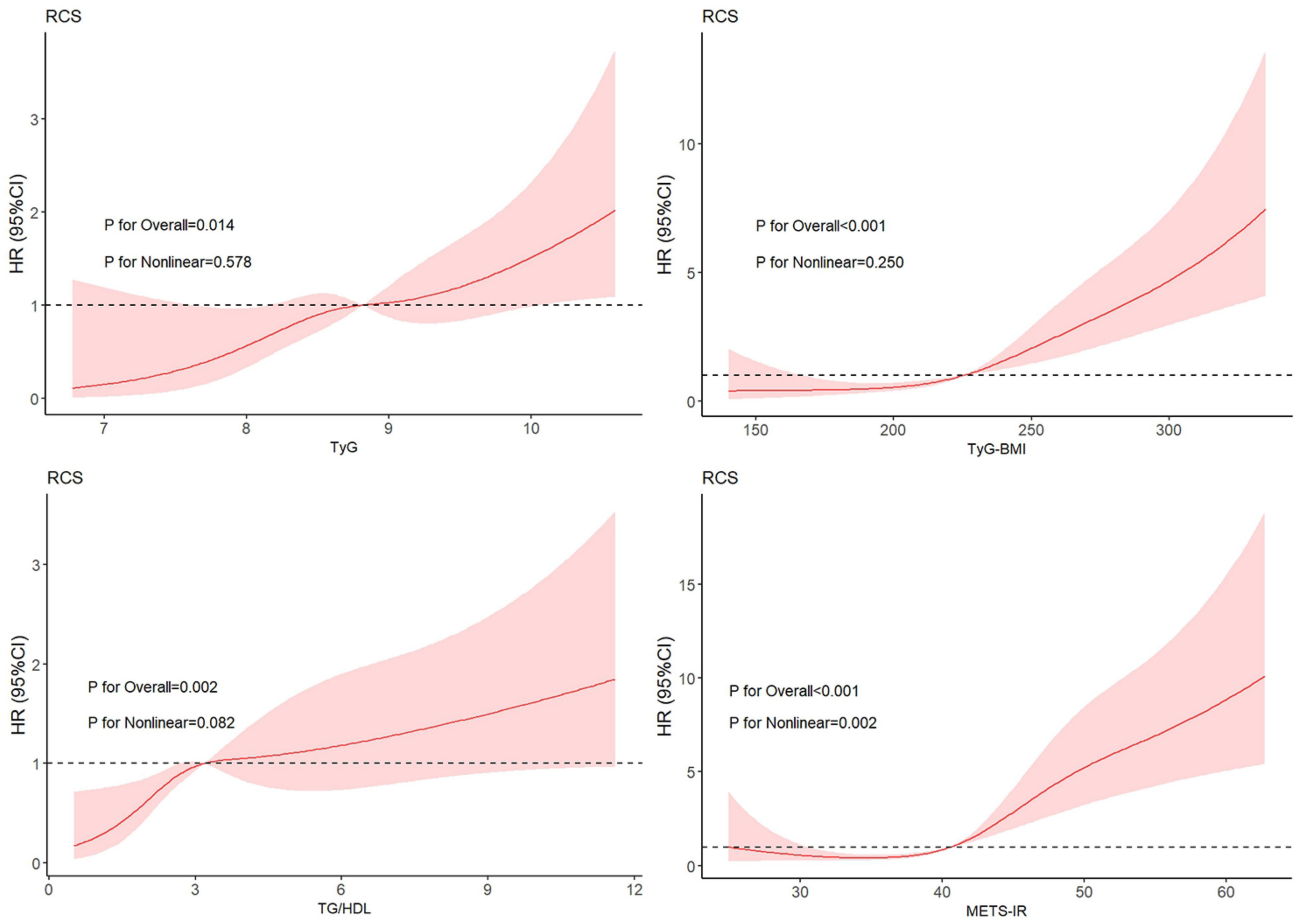
Restricted cubic spline curves for MACEs by non-insulin-based IR indices.

All four IR indices were significantly associated with MACEs across unadjusted and adjusted models (Table 2). After full adjustment for age, sex, smoking, diabetes, Gensini score, and antidiabetics, METS-IR demonstrated the highest HR (1.104, 95% CI: 1.080–1.129, *P* < 0.001). Schoenfeld residual tests confirmed that the proportional hazards assumption was satisfied for all four models (all global *P* > 0.70; Supplementary Table S1), and all indices remained significant after Bonferroni correction for multiple comparisons (*α* = 0.0125). RCS analysis revealed linear associations for TyG-BMI (*P* for non-linear = 0.250) and TG/HDL-C (*P* for non-linear = 0.082), but non-linear relationships for TyG (*P* for non-linear = 0.578) and METS-IR (*P* for non-linear = 0.002) (Figure 2), suggesting differences in their predictive patterns.

**Table 2:** Association between non-insulin-based IR indices and MACEs.

IR index	Model 1		Model 2		Model 3	
	HR (95%CI)	*P* value	HR (95%CI)	*P* value	HR (95%CI)	*P* value
TyG index	1.836 [1.411, 2.391]	<0.001	1.875 [1.445, 2.434]	<0.001	1.627 [1.228, 2.145]	<0.001
TyG-BMI	1.017 [1.013, 1.021]	<0.001	1.017 [1.013, 1.021]	<0.001	1.019 [1.014, 1.023]	<0.001
TG/HDL-C	1.148 [1.077, 1.223]	<0.001	1.151 [1.080, 1.226]	<0.001	1.127 [1.055, 1.195]	<0.001
METS-IR	1.104 [1.082, 1.128]	<0.001	1.102 [1.080, 1.226]	<0.001	1.104 [1.080, 1.129]	<0.001

Model 1: Unadjusted.

Model 2: Adjusted for age and sex.

Model 3: Adjusted for age, sex, smoking, diabetes, Gensini score, Antidiabetics.

### 3.3 Incremental predictive value of IR indices

**Figure 3:**
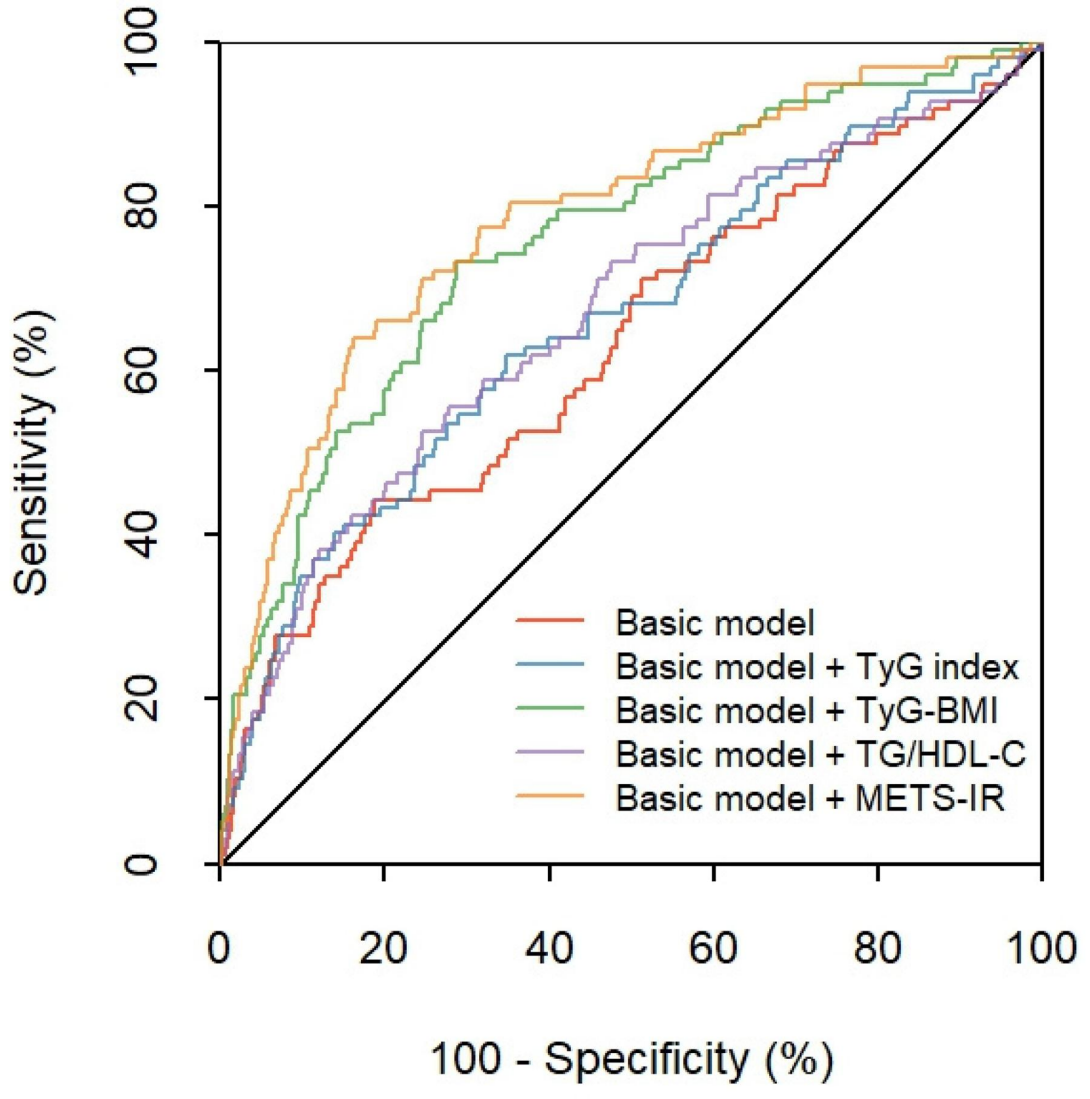
ROC curves of predicting MACEs by non-insulin-based IR indices.

ROC analysis (Figure 3, Table 3) showed that the baseline model (age, sex, smoking, diabetes, Gensini score, antidiabetics) had an AUC of 0.631. Adding IR indices improved AUCs to 0.662 (TyG index), 0.759 (TyG-BMI), 0.671 (TG/HDL-C), and 0.785 (METS-IR) (all *P* < 0.05). Incremental prognostic performance was further assessed using C-statistic, NRI, and IDI (Table 4). METS-IR demonstrated the highest incremental value with significant improvements in both IDI (0.082, *P* < 0.001) and NRI (0.356, *P* < 0.001). Notably, while TyG index showed a statistically significant association with MACEs, its negative NRI (−0.005, *P* = 0.358) suggests limited improvement in individual risk reclassification beyond the baseline model.

**Table 3:** ROC curve analysis for predicting MACEs.

Model	AUC (95%*CI*)	Cutoff value	Sensitivity/%	Specificity/%	*P* value
Basic model	0.631 [0.568, 0.694]	–	–	–	Reference
+TyG index	0.662 [0.601, 0.723]	8.38	41.23	85.50	0.035
+TyG-BMI	0.759 [0.706, 0.812]	265.35	74.23	74.23	<0.001
+TG/HDL-C	0.671 [0.611, 0.732]	4.27	55.67	71.88	0.014
+METS-IR	0.785 [0.742, 0.828]	48.01	78.50	74.20	<0.001

Basic model: age, sex, smoking, diabetes, Gensini score, Antidiabetics.

**Table 4:** Incremental prognostic value of adding IR indices to the baseline model.

Model	C-statistic (95% CI)	*P* value	IDI (95% CI)	*P* value	NRI (95% CI)	*P* value
Basic model	0.625 [0.563, 0.688]	Reference	Reference		Reference	
+TyG index	0.666 [0.607, 0.725]	<0.001	0.013 [−0.003, 0.055]	0.189	−0.005 [−0.175, 0.132]	0.358
+TyG-BMI	0.757 [0.704, 0.811]	<0.001	0.049 [0.000, 0.120]	0.050	0.249 [0.095, 0.376]	0.020
+TG/HDL-C	0.673 [0.615, 0.730]	<0.001	0.017 [−0.005, 0.053]	0.179	0.123 [−0.048, 0.256]	0.229
+METS-IR	0.779 [0.727, 0.831]	<0.001	0.082 [0.032, 0.149]	<0.001	0.356 [0.204, 0.485]	<0.001

Basic model: age, sex, smoking, diabetes, Gensini score, Antidiabetics.

In sensitivity analyses adjusting for additional medications, the HRs for all four IR indices remained materially unchanged (Supplementary Table S2), suggesting that medication use did not substantially confound the observed associations.

### 3.4 Subgroup analysis

**Figure 4:**
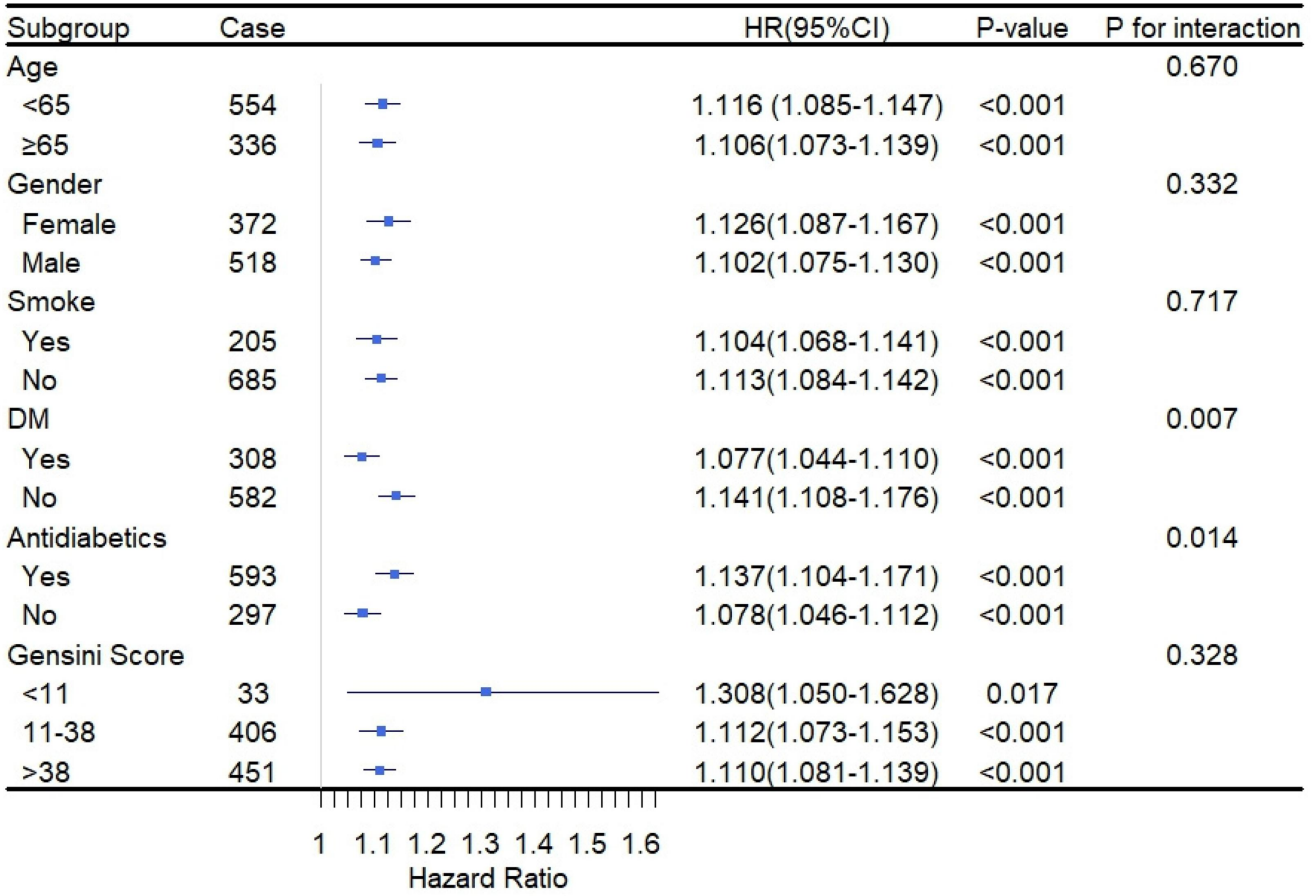
Subgroup analysis of METS-IR and MACEs.

Subgroup analyses demonstrated a consistent positive association between METS-IR and MACEs (Figure 4). Significant interactions were observed for diabetes (*P* for interaction = 0.007) and antidiabetics (*P* for interaction = 0.014), with stronger associations in patients without diabetes and those not using antidiabetics. No significant interactions were found for age, sex, smoking, or Gensini score (*P* for interaction: 0.670, 0.332, 0.717, and 0.328, respectively). These findings suggest that while the prognostic value of METS-IR is generally robust, its magnitude may be attenuated in patients with diabetes or those on antidiabetics therapy.

### 3.5 Survival analysis

**Figure 5:**
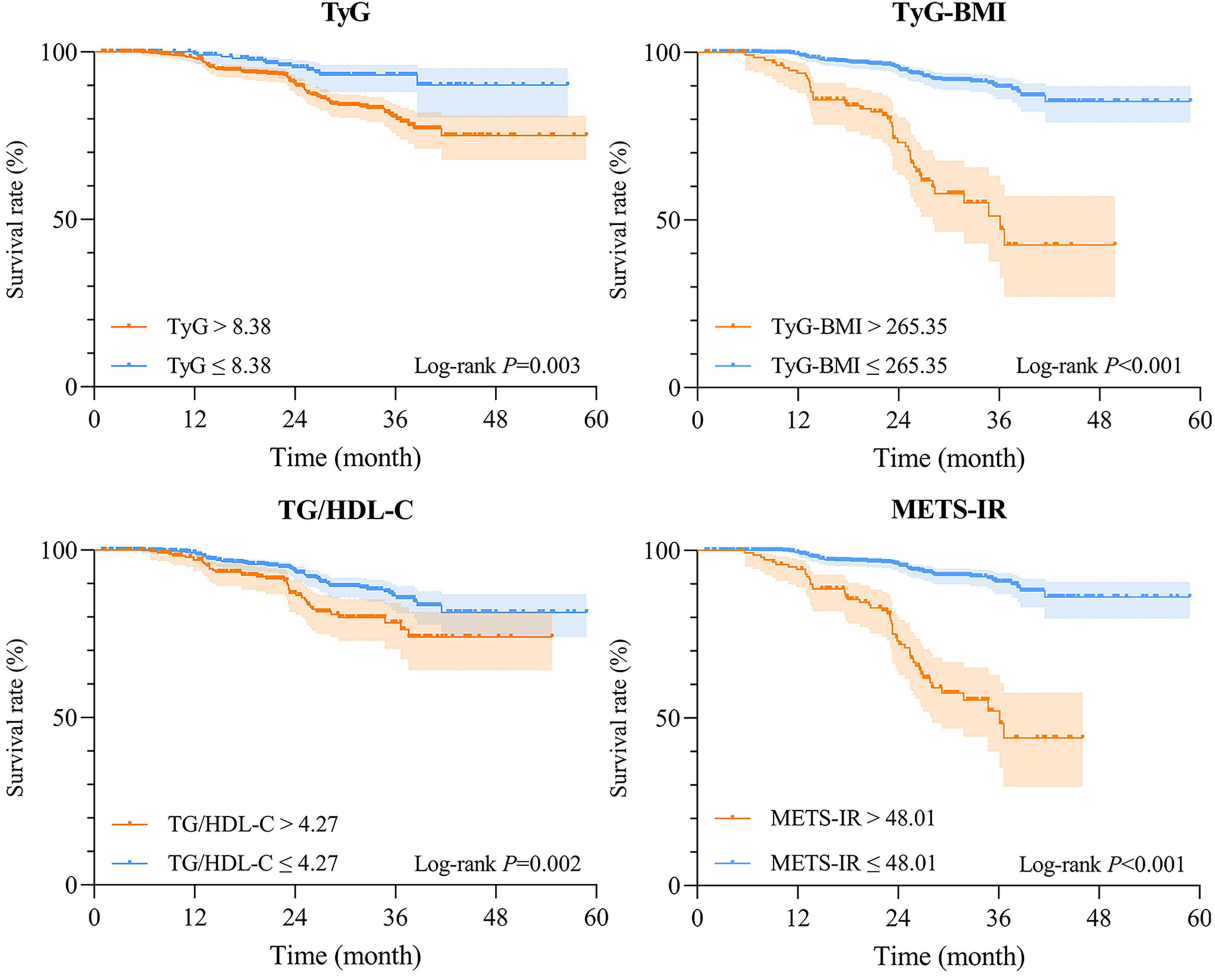
Kaplan–Meier survival curves for MACEs by IR index groups.

Over a median follow-up of 26.5 months (IQR: 19.7–33.0), 97 MACEs occurred. Patients were stratified into high- and low-IR groups based on optimal Youden index-derived cut-offs. Kaplan–Meier analysis showed significantly higher MACEs rates in the high-IR groups for all indices (log-rank *P* < 0.01, Figure 5), further supporting the association between elevated IR and increased cardiovascular risk.

## 4 Discussion

In this cohort study of 890 patients with newly diagnosed CAD, we evaluated the prognostic performance of four non-insulin-based IR indices for predicting MACEs. All four indices—TyG, TyG-BMI, TG/HDL-C, and METS-IR—were independently associated with MACEs after multivariable adjustment. Among them, METS-IR demonstrated the highest incremental prognostic value across all four evaluative metrics (AUC, C-statistic, IDI, and NRI). Kaplan-Meier analysis further confirmed that higher IR levels were associated with significantly increased MACE risk. These findings underscore the potential clinical utility of IR indices, particularly METS-IR, as practical tools for risk stratification in patients with newly diagnosed CAD.

IR, a hallmark of of metabolic dysregulation, promotes atherosclerosis through multiple interconnected pathways, including chronic inflammation, endothelial dysfunction, and atherogenic dyslipidemia (16–18). Hyperinsulinemia and hyperglycemia accelerate vascular smooth muscle proliferation, oxidative stress, and plaque destabilization (19). The lipid profile characteristic of IR—elevated TG, LDL-C, and reduced HDL-C—further promotes foam cell formation and plaque vulnerability (20, 21). IR also contributes to hypertension and hypercoagulability, collectively increasing cardiovascular risk (22).

Recent years have seen the development several practical IR indices that utilize routine metabolic parameters, circumventing the need for insulin measurement (7–10). The TyG index has been extensively studied and is significantly associated with all-cause and cardiovascular mortality in the general population, particularly among individuals under 65 years of age (23). In patients with established CAD, the TyG index is positively correlated with future cardiovascular events (24). However, the relationship may be more complex in certain populations; Zhang et al. identified a U-shaped association between baseline TyG index and mortality in patients with diabetes or prediabetes and cardiovascular disease (25). TyG-BMI, which combines the TyG index with BMI, better captures the impact of obesity and fat distribution on metabolic status. Some studies have reported stronger correlation with coronary atherosclerosis burden and prognosis compared with TyG alone (26, 27). The TG/HDL-C ratio, a traditional marker of lipid disorders, has been consistently associated with CAD severity and cardiovascular events (28, 29). This study further supports this conclusion. However, the gain of TG/HDL-C ratio in fine individual risk reclassification remains limited. Our findings corroborate this association, yet the ratio's modest gains in reclassification metrics (NRI = 0.123, *P* = 0.229) indicate that it may serve best as an adjunctive tool for lipid management rather than a primary risk stratification instrument.

METS-IR integrates glucose, lipid, and adiposity measures into a multidimensional score, offering a more comprehensive assessment of metabolic risk (10). It correlates strongly with metabolic syndrome components and has been shown to predict diabetes and cardiovascular events in diverse populations (30–33). In this study, METS-IR showed the highest prognostic utility among the four indices, with the largest AUC (0.785), C-statistic (0.779), IDI (0.082), and NRI (0.356). These findings align with previous reports suggesting that METS-IR may have superior discrimination ability for predicting CAD presence and severity compared with other non-insulin-based IR indexes (30). The superiority of METS-IR likely stems from its comprehensive capture of multiple metabolic risk dimensions—glucose, lipid, and adiposity—rather than relying on a single metabolic pathway. Furthermore, the non-linear association observed between METS-IR and MACEs (*P* for nonlinear = 0.019), coupled with its significant interaction with diabetic status (*P* for interaction = 0.007), suggests that METS-IR may be particularly relevant in dysglycemic populations, where metabolic dysregulation is more pronounced.

This study has several limitations. First, the single-center retrospective design and the restriction to an East Asian cohort limit the generalizability of our findings to other populations and settings. Second, although we adjusted for RAS inhibitors, beta-blockers, and CCBs in sensitivity analyses, we could not account for novel glucose-lowering agents such as SGLT2 inhibitors or GLP-1 receptor agonists, which may influence IR and MACEs. Third, the use of baseline IR indices only precludes assessment of dynamic changes in insulin resistance over time and their prognostic implications. Fourth, while LASSO penalized regression (EPV ≈ 13.9) and Firth penalized estimation were implemented to mitigate concerns about overfitting, residual overfitting cannot be completely ruled out. Fifth, although Gensini score was included as a covariate, we acknowledge that this may represent overadjustment if insulin resistance mediates the relationship between metabolic dysfunction and CAD severity; this remains to be addressed in future studies. Despite these limitations, the consistent findings across multiple analytical approaches support the robustness of our conclusions.

## 5 Conclusion

This study demonstrates that the TyG index, TyG-BMI, TG/HDL-C ratio, and METS-IR are all independently associated with MACEs in patients with newly diagnosed CAD. Among them, METS-IR showed the strongest incremental prognostic value. These findings support the role of IR in CAD progression and highlight the potential of METS-IR as a practical tool for risk stratification and personalized management.

## Data Availability

The raw data supporting the conclusions of this article will be made available by the authors, without undue reservation.
