# Supplementary material for: Prognostic value of non-insulin-based insulin resistance indices in patients with newly diagnosed coronary artery disease: a cohort study
Source: Front Cardiovasc Med. 2026 Jul 22;13:1863508. doi: 10.3389/fcvm.2026.1863508 (PMC13439648; doi:10.3389/fcvm.2026.1863508)
Supplement: Supplementary file 1 [file Table1.docx]

**Supplementary Table S1.** Proportional hazards assumption test (Schoenfeld residual tests)

| **IR index** | **Global χ²** | ***df*** | **Global P‑value** |
| --- | --- | --- | --- |
| TyG index | 2.978 | 7 | 0.887 |
| TyG-BMI | 4.512 | 7 | 0.719 |
| TG/HDL-C | 2.782 | 7 | 0.904 |
| METS-IR | 2.159 | 7 | 0.951 |

Data are presented as χ² and P values for the global test from Schoenfeld residual tests.

All global *P* > 0.70, indicating no violation of the proportional hazards assumption for all four models.
