# Supplementary material for: Prognostic value of non-insulin-based insulin resistance indices in patients with newly diagnosed coronary artery disease: a cohort study
Source: Front Cardiovasc Med. 2026 Jul 22;13:1863508. doi: 10.3389/fcvm.2026.1863508 (PMC13439648; doi:10.3389/fcvm.2026.1863508)
Supplement: Supplementary file 2 [file Table2.docx]

**Supplementary Table S2.** Sensitivity analysis adjusting for medications

| **IR Index** | **Primary model** | | **Drug‑adjusted model** | | |
| --- | --- | --- | --- | --- | --- |
|  | **HR (95%CI)** | ***P* value** | **HR (95%CI)** | ***P* value** | |
| TyG index | 1.627 [1.228, 2.145] | <0.001 | 1.603 [1.213, 2.120] | | <0.001 |
| TyG-BMI | 1.019 [1.014, 1.023] | <0.001 | 1.018 [1.014, 1.023] | | <0.001 |
| TG/HDL-C | 1.127 [1.055, 1.195] | <0.001 | 1.127 [1.058, 1.202] | | <0.001 |
| METS-IR | 1.104 [1.080, 1.129] | <0.001 | 1.105 [1.080, 1.129] | | <0.001 |

Primary model adjusted for age, sex, smoking, diabetes, Gensini score.

Drug‑adjusted model further adjusted for RAS inhibitors, beta‑blockers, CCBs, Antidiabetics.
